# Metagenomic analysis of Ancient Egyptian canopic jars

**DOI:** 10.1002/ajpa.24600

**Published:** 2022-08-05

**Authors:** Enrique Rayo, Judith Neukamm, Nadja Tomoum, Patrick Eppenberger, Abagail Breidenstein, Abigail S. Bouwman, Verena J. Schuenemann, Frank J. Rühli

**Affiliations:** ^1^ Institute of Evolutionary Medicine University of Zurich Zurich Switzerland

**Keywords:** ancient DNA, Ancient Egypt, canopic jars, metagenomics

## Abstract

Ancient Egyptian remains have been of interest for anthropological research for decades. Despite many investigations, the ritual vessels for the internal organs removed during body preparation—liver, lungs, stomach, and intestines, of Egyptian mummies are rarely used for palaeopathological or medical investigations. These artifacts, commonly referred to as canopic jars, are the perfect combination of cultural and biological material and present an untapped resource for both Egyptological and medical fields. Nevertheless, technical challenges associated with this archeological material have prevented the application of current ancient DNA techniques for both the characterization of human and pathogenic DNA. We present shotgun‐sequenced metagenomic profiles and ancient DNA degradation patterns from multiple canopic jars sampled from several European museum collections and enumerate current limitations and possible solutions for the future analysis of similar material. This is the first‐ever recorded evidence of ancient human DNA found in Ancient Egyptian canopic jars and the first associated metagenomic description of bacterial taxa in these funerary artifacts.

**Objectives:**

In this study, our objectives were to characterize the metagenomic profile of the Ancient Egyptian funerary vessels known as canopic jars to retrieve endogenous ancient human DNA, reconstruct ancient microbial communities, and identify possible pathogens that could shed light on disease states of individuals from the past.

**Methods:**

We applied ancient DNA techniques on 140 canopic jars to extract DNA and generate whole‐genome sequencing libraries for the analysis of both human and bacterial DNA. The samples were obtained from museum collections in Berlin (DE), Burgdorf (DE), Leiden (NE), Manchester (UK), Munich (DE), St. Gallen (CH), Turin (IT), and Zagreb (HR).

**Results:**

Here we describe the first isolated DNA from the Egyptian artifacts that hold human viscera. No previous work was ever conducted on such material, which led to the first characterization of human DNA from Ancient Egyptian canopic jars and the profiling of the complex bacterial composition of this highly degraded, challenging, organic material. However, the DNA recovered was not of enough quality to confidently characterize bacterial taxa associated with infectious diseases, nor exclusive bacterial members of the human microbiome.

**Discussion:**

In summary, we present the first genomic survey of the visceral content of Ancient Egyptian funerary artifacts and demonstrate the limitations of current molecular methods to analyze canopic jars, such as the incomplete history of the objects or the presence of uncharacterized compounds that can hamper the recovery of DNA. Our work highlights the main challenges and caveats when working with such complicated archeological material – and offers sampling recommendations for similarly complex future studies, such as incrementing the amount of starting material and sampling from the less exposed parts of the jar content. This is the first‐ever recorded evidence of ancient human DNA found in Ancient Egyptian canopic jars, and our results open new avenues in the study of neglected archeological artifacts.

## 1 INTRODUCTION

Canopic jars are funerary vessels for the principal viscera of Ancient Egyptian mummies, although other objects in different forms and shapes are also designated more generally as “canopic equipment” designation (Ikram & Dodson, 1998). The first canopic containers were developed circa 2600 BC (4th Dynasty, Old Kingdom), designated jars to preserve four particular organs for the afterlife: the liver, the lungs, the stomach, and the intestines. Despite their presence in the archeological record, the ritual vessels for the internal organs removed during body preparation remain rarely used for palaeopathological or medical investigations (Habicht et al., 2013; Senti et al., 2018; Walker et al., 1987). Since most pathogens and chronic diseases are localized in the internal organs, these artifacts are the perfect combination of cultural and biological material and present an untapped resource for medical investigations of Ancient Egypt (Galassi et al., 2017; Rühli et al., 2015; Senti et al., 2018). Radiological (Eppenberger et al., 2018) and toxicological data (Brockbals et al., 2018) from these selected canopic jars have been published, but genetic analyses are still not available for these artifacts. Such ancient DNA analysis would help elucidate some of the recent questions concerning canopic jars, such as confirming the presence of actual human tissue, characterizing the type of tissue present, identifying the presence of commensal or pathogenic bacteria, and gathering further information about organic components that may be present in the jars.

Ancient DNA analysis is now a standard tool in the repertoire of archeological studies. However, Ancient Egyptian material presents several limitations due to Egypt's arid climate and high humidity levels in tomb microenvironments, a combination of factors that negatively impact DNA preservation (Gilbert et al., 2005). In addition, several concerns have been raised regarding modern contamination of Ancient Egyptian samples (Lorenzen & Willerslev, 2010), even for those studies relying on high‐throughput technologies (Khairat et al., 2013). For these reasons, any molecular findings that claimed the retrieval of ancient DNA from Ancient Egyptian remains (Hawass et al., 2010) were received with skepticism (Timmann & Meyer, 2010). It was not until recently that the latest contamination procedures and high‐throughput sequencing techniques have been successfully applied to Egyptian mummies (Loreille et al., 2018; Neukamm et al., 2020; Schuenemann et al., 2017), providing a proof of concept that both endogenous human, bacterial, and viral aDNA can be validated in samples from Ancient Egyptian materials.

Here we present an unprecedented paleogenomics analysis of the content of Ancient Egyptian canopic jars. Our goal was to characterize the metagenomic profile of the contents of the jars to retrieve endogenous ancient human DNA, reconstruct microbial communities, and identify possible pathogens. By exploring non‐conventional archeological sources to analyze ancient DNA, we aim to make recommendations for high‐throughput sequencing library preparation and data analysis when working with such materials.

## 2 MATERIALS AND METHODS

**FIGURE 1:**
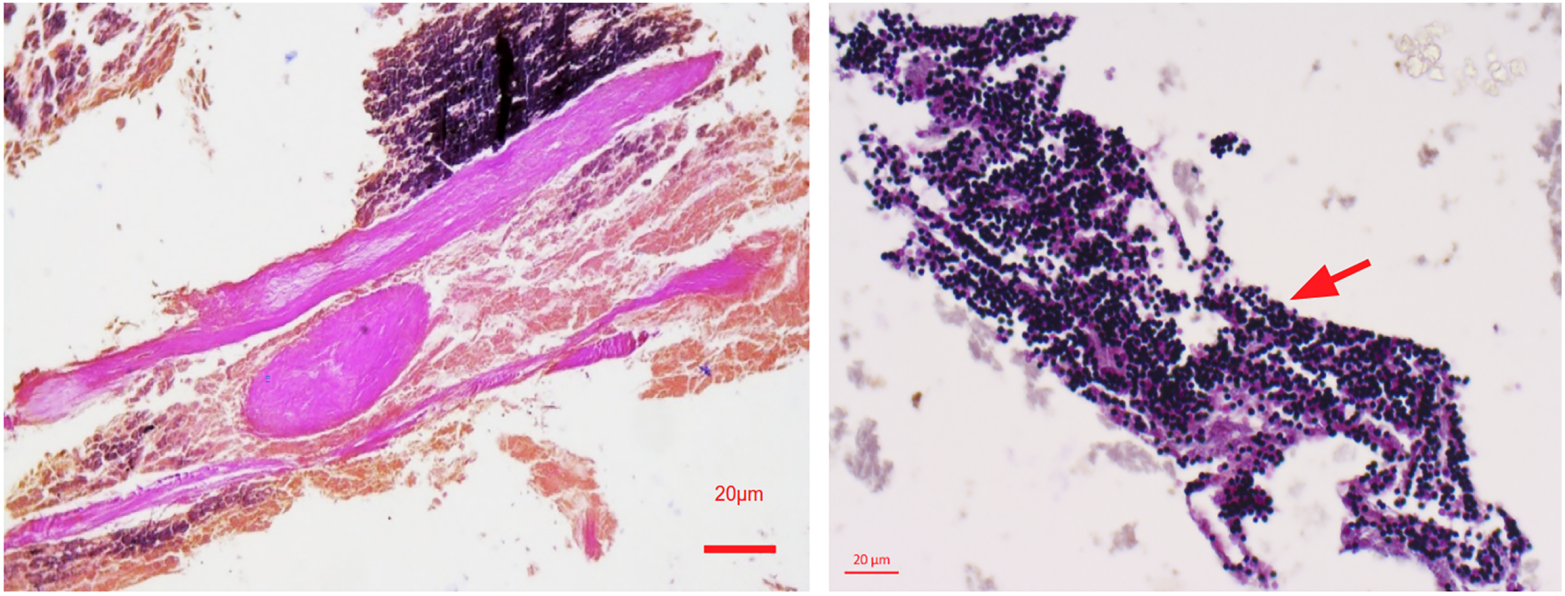
Histology slides from the Zagreb samples, scaled. Left, sample 607 with Periodic acid–Schiff stain (PAS) staining showing fibrous structures, most likely connective tissue or muscle. Right, sample 622‐1 with Gram‐stained conglomerates of spherical gram‐positive structures (arrow)

Museum collections from Berlin (DE), Burgdorf (DE), Leiden (NE), Manchester (UK), Munich (DE), St. Gallen (CH), Turin (IT), and Zagreb (HR), provided access to 140 canopic jars for potential sampling; a total of 93 jars were selected for ancient DNA screening and 50–500 mg of starting material was sampled (Table S1). After testing various extraction methods that showed no distinctive success when retrieving DNA (SI.1), we opted for a standard extraction method based on Dabney et al. with slight modifications (Dabney et al., 2013). For 41 samples, sequencing libraries were built and sequenced on Illumina sequencing platforms (Kircher et al., 2012; Meyer & Kircher, 2010). Based on histological observations (Figure 1, Figures S1–S4), a subset of 13 samples with possible preservation of soft tissues was selected for further processing. To optimize the recovery of short fragments of human DNA, we prepared single‐stranded DNA libraries from these samples (Gansauge & Meyer, 2019) and performed an enrichment step using a hybridization capture protocol with custom biotinylated baits for human mitochondrial DNA (mtDNA) (Furtwängler et al., 2020; Maricic et al., 2010). Sample contexts, data availability, and methods used in this study are detailed in SI.1 and SI.2.

## 3 RESULTS AND DISCUSSION

**FIGURE 2:**
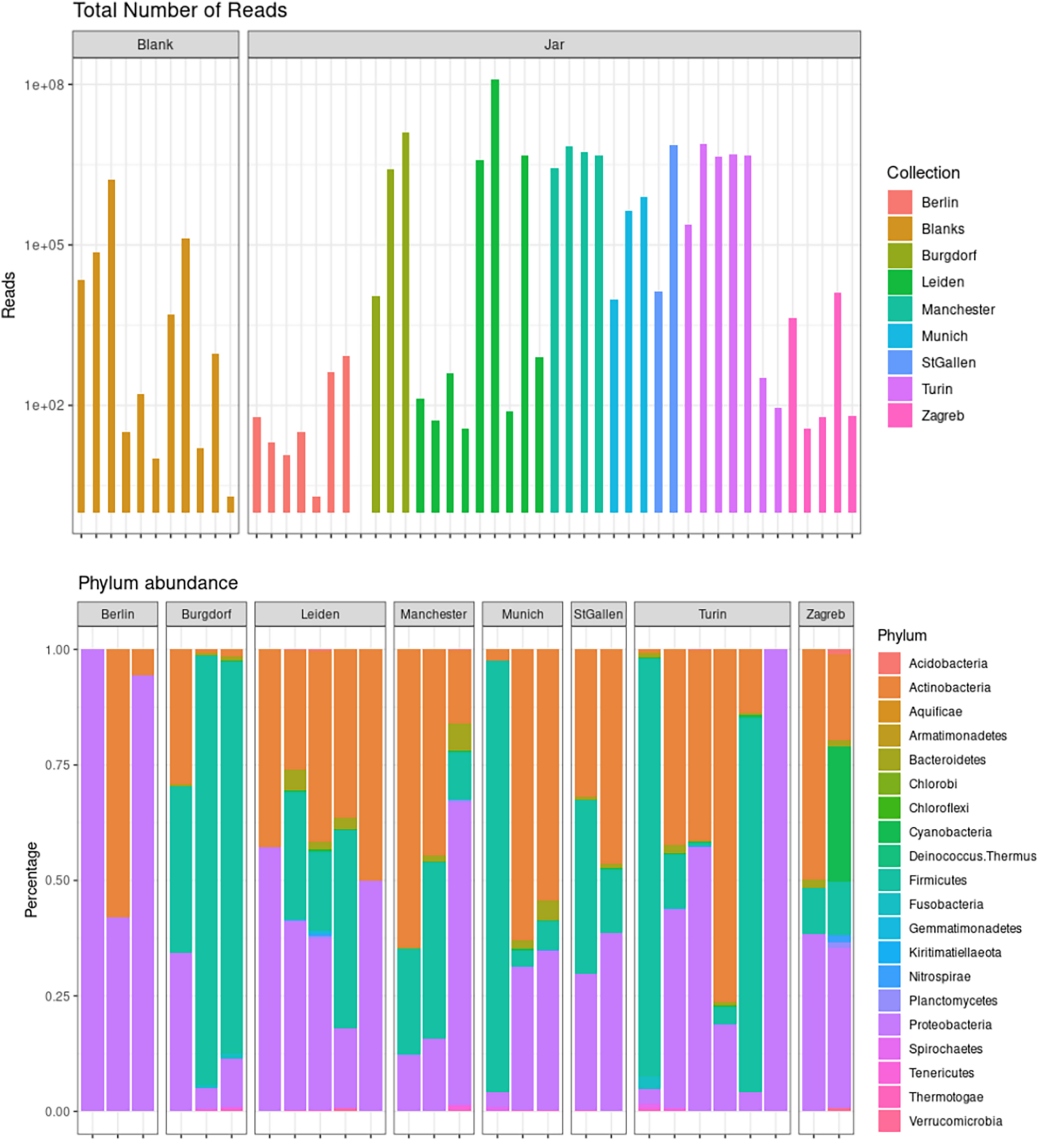
Top: Total number of sequencing reads present in each sample, grouped by negative controls (Blanks) and sample (Jar); colors correspond to the collection. Bottom: Abundance of phyla present in each sample expressed in percentage, grouped by collection

The initial shotgun sequencing using double‐stranded libraries of 41 samples produced a low output for most samples, from zero reads to less than 1000 reads for 20 of the samples. Only 14 samples surpassed one million reads. This generally low amount of reads retrieved after initial sequencing (Figure 2) was the first indication of low DNA preservation and potential inhibition. For the initial assessment of ancient DNA preservation in the samples, we screened shotgun sequencing outputs for reads that could be mapped against the human mtDNA. This screening for mtDNA produced no results in all samples. To increase the endogenous content of human mtDNA, we performed an in‐solution hybridization capture with DNA baits on the samples that presented an identification of histological remains. After enrichment and deeper sequencing, these 14 selected samples from Berlin and Zagreb (Table S2) gave a low amount of reads mapping to human mtDNA (less than 1000 reads) (Table S2) except for three samples: Zagreb 607 (with 8081 reads) (Figure S5), Berlin AM7170/3 (4398 reads) (Figure S6), and Berlin AM7179 (3142 reads) (Figure S7). However, damage frequencies characteristic of aDNA were quite low (3% for 607, 9% for AM7170/3, and 8% for AM7179). When consensus sequences were assigned a haplogroup (Renaud et al., 2015; Weissensteiner et al., 2016), Zagreb 607 was characterized as the European haplogroup H (92% quality, 0.01 estimated contamination), and sample Berlin AM7179 was assigned to haplogroup R0a1 (70% quality, 0.01 estimated contamination), frequent in modern Middle‐East populations. Berlin AM7170/3 could not be assigned to a haplogroup. The haplogroup H has been reported to be present in both ancient and modern Egyptian populations in variable degrees but is more frequent in modern populations, while R0 and its subgroups are more frequent in ancient populations, although also still present in modern‐day Egyptian groups (Drosou et al., 2020; Pagani et al., 2015; Schuenemann et al., 2017) Taken together, the levels of DNA damage observed are within the range of previously described molecular profiles of samples from Ancient Egyptian mummies (Neukamm et al., 2020; Schuenemann et al., 2017); however, the risk of fractional contamination by modern DNA should not be ruled out—especially considering that the samples are stored in European collections, and the outcomes of this work should be taken with caution.

When screened for metagenomic bacterial markers, the jars did not present a specific signature based on collection or tissue, but rather a sample‐dependent profile dominated by Proteobacteria, Acidobacteria, or Firmicutes (Figure 2). Most bacterial families present in the jars are widespread in most environments (Dicks & Endo, 2014; Mandic‐Mulec et al., 2015; Octavia & Lan, 2014; Voronina et al., 2015), and members of bacteria that are nearly ubiquitous and very resistant to heat, desiccation, and toxic chemicals, such as *Clostridium*, *Streptomyces*, *Burkholderia* or *Virgibacillus* (SI.3, Figure S8). This suggests that most bacteria in these samples were likely to be present due to environmental contamination, a problem that has been raised to attention recently in the analysis of ancient microbiomes (Farrer et al., 2021) but that is often overlooked in most ancient metagenomic analysis. Taxonomic characterization was more difficult at the genus level. Some samples (mainly from Leiden, Berlin, and Zagreb) had no definitory resolution to discern between widespread and human‐associated bacteria (e.g., *Mycobacterium*, *Lactobacillus*). Most of the genera identified are present in most environments and some are known to be resistant to extreme saline conditions (e.g., *Oceanobacillus iheyensis*) (SI.3), which may be related to the materials used during the mummification process. We surveyed for bacterial species that are isolated primarily from the human gut microbiome of modern and past populations (Lloyd‐Price et al., 2016; Moeller et al., 2014; Rampelli et al., 2021; Schnorr et al., 2014), and identified members of the genera *Alistipes*, *Bifidobacterium*, *Desulfovibrio*, *Faecalibacterium*, and *Klebsiella*, with damage profiles also in the ranges of 7%–12% (Table S3). However, these taxa were not present equally in all samples nor presented enough coverage to ascertain their role as commensal bacteria of the ancient microbiome. The number of reads (Figure 2) was insufficient to discern with confidence other major taxonomic groups, such as fungi or viruses (SI.2).

Overall, surveying canopic jars as a source of human and bacterial ancient DNA demonstrated several limitations. First, most of the supposedly unopened jars had no content, having been thoroughly emptied at some point between their discovery and sampling for the study. Out of the 140 vessels to which we had access, only 61 held putatively organic material suitable for molecular testing. Second, for the few jars with suitable content, it is unclear whether they contained soft tissues. Previous studies relying on volumetric calculations from CT/MRI scans proved these artifacts are physically not large enough to hold entire human organs (Eppenberger et al., 2018), which substantially reduced the theoretical possibility of recovering soft tissue remains for molecular studies. Finally, the building of DNA sequencing libraries from the sampled material was somewhat inefficient. Only 41 of these 93 samples generated DNA libraries with enough quality to warrant sequencing. The yield of those sequenced libraries was quantitatively similar to the yield of the negative control extracts. Although most methods to extract ancient DNA are designed to remove common inhibitors, for example, humic acids (Matheson et al., 2010), this suggests the presence of inhibitory substances that interfere with steps within the molecular pipeline—namely, library preparation, barcoding, or amplification. It is also important to consider the possibility of very poor DNA preservation in these presumed soft tissue samples, a pattern previously established for Ancient Egyptian material (Schuenemann et al., 2017). The exact nature of the embalming agents used for organ preservation within the canopic jars is unclear, although recent metabolomics studies suggested the presence of several plant components (Brockbals et al., 2018). With this composition in mind, we tested several extraction protocols designed to remove plant‐based inhibitors (SI.1), yet we did not observe any increase in DNA yield. This suggests the presence of uncharacterized inhibitory compounds in the contents of canopic jars. These results highlight the need for more extensive analysis to interpret the chemical constitution of canopic jars, as previously noted (Brockbals et al., 2018) to elucidate whether our results are due to chemical interference or simply a DNA preservation issue. From one of our previous experimental mummification studies (Öhrström et al., 2020), we know that out of the four types of viscera typically encountered in ancient Egyptian canopic jars (lung, liver, stomach, and intestines), the liver showed the best preservation of cellular structures histologically. In contrast, the lung underwent substantial tissue alterations in size and structure, which was equally reflected in the degree of DNA preservation, being the lowest in the lung specimen. Consequently, tissue density appeared to play an essential role in histological preservation and DNA recovery.

The above factors may explain why we observe very low amounts of ancient DNA in material sampled from the canopic jars. Bacterial species identified in these canopic jars were predominantly widespread microorganisms that thrive in harsh, low‐nutrient environments (e.g., *Clostridium* spp., and *Streptomyces* spp.), and more importantly, their likely introduction in the samples cannot be pinpointed to a specific moment in the history of the jars. Taking into consideration microbial contaminants that are also of ancient origin adds another level of complexity to the study of poorly preserved communities, such as in the case of canopic jars, and it is an issue that should be addressed more frequently in ancient microbiome studies. As for the assumption of human tissue presence, no bacteria known to be exclusive to the human microbiome were detected based on the current knowledge on commensal microbial communities, nor was there enough resolution to detect bacterial taxa associated with human infectious diseases. Sequencing reads from two samples could be mapped against human mitochondrial DNA, although contamination from unknown human sources in the recent past should not be disregarded in a similar manner to that of exogenous microbe contamination mentioned above.

## 4 CONCLUSIONS

In summary, we present the first genomic survey of the visceral content of Ancient Egyptian funerary artifacts and demonstrate the limitations of current molecular methods to analyze canopic jars. The often‐obscure history of canopic jars—no recorded history of the objects, decontextualized, unknown content replacement—calls for a multidisciplinary, comprehensive assessment of the objects before sampling. Whenever possible, a higher amount of starting material, ideally taken from the deeper, less exposed areas of the jar, should be collected to maximize tissue recovery and facilitate parallel analysis of the sample (e.g., histology, radiocarbon dating, metabolomics, and metagenomics). Assessing the suitability of archeological material in advance is necessary to reduce these associated time and cost constraints when analyzing samples from canopic jars. Future studies on canopic jars and similarly complex ancient remains will benefit from a systematic approach to addressing the limitations identified. Despite these mentioned drawbacks, we were able to characterize ancient human DNA and ancient bacteria DNA from the content of Egyptian funerary vessels.

## AUTHOR CONTRIBUTIONS

**Enrique Rayo:** Conceptualization (supporting); formal analysis (lead); investigation (lead); visualization (lead); writing – original draft (lead). **Judith Neukamm:** Software (supporting); writing – review and editing (supporting). **Nadja Tomoum:** Validation (supporting); writing – review and editing (supporting). **Patrick Eppenberger:** Methodology (supporting); writing – review and editing (supporting). **Abagail Breidenstein:** Writing – review and editing (supporting). **Abigail S. Bouwman:** Conceptualization (equal); funding acquisition (equal); supervision (equal); writing – review and editing (supporting). **Verena J. Schuenemann:** Supervision (equal); writing – review and editing (supporting). **Frank J. Ruhli:** Conceptualization (lead); funding acquisition (equal); supervision (equal); writing – review and editing (supporting).

## CONFLICT OF INTEREST

The authors declare that we have no conflict of interest.

## Supporting information


**Appendix S1** Supporting Information.

## Data Availability

The sequencing data used for this study can be found in the Sequence Read Archive (SRA) under the BioProject ID: PRJNA855637, with the scheduled release on 2022‐09‐30.
