# Supplementary material for: Metagenomic analysis of Ancient Egyptian canopic jars
Source: Am J Biol Anthropol. 2022 Aug 5;179(2):307–13. doi: 10.1002/ajpa.24600 (PMC9804471; doi:10.1002/ajpa.24600)
Supplement: Supplementary file 1 — Appendix S1 Supporting Information. [file AJPA-179-307-s001.docx]

# **Metagenomic profile of Ancient Egypt Canopic Jars - Supplementary Information**

Enrique Rayo ^1,*^, Judith Neukamm ^1^, Nadja Tomoum ^1^, Patrick Eppenberger ^1^, Abagail Breidenstein ^1,^ Abigail S. Bouwman ^1,#,*^, Verena J. Schuenemann ^1,#^, Frank J. Rühli ^1,#^

1. Institute of Evolutionary Medicine, University of Zurich, Zurich, Switzerland

#. Jointly supervised this study

*. Corresponding authors

## **Supplementary note 1 - Laboratory workflow**

### **Samples used in this study**

Canopic containers were developed to preserve four particular organs for the afterlife: the liver, the lungs, the stomach, and the intestines (Michael E. Habicht, Bouwman, and Rühli 2013; Senti et al. 2018). Canopic jars with human-headed lids represented protection deities - the four sons of Horus protected the organs: Amset protected the liver, Hapy the lungs, Duamutef the stomach, and Qebekhsenuef the intestines. From the Ramesside period onwards (19th and 20th Dynasty) the Canopic lids were depicted with the heads of the protection deities: Amsety remained human-headed, Hapy was depicted as a baboon, Duamutef as a jackal, and Qebekhsenuef as a falcon, although the association between Duamutef and Qebehsenuef was not clear, Duamutef being represented with the falcon head and Qebehsenuef depicted as a jackal (Gadalla 2001).

The Canopic Jar Project was granted access to a total of 140 jars from different collections and museums all over the world: Zentrales Zwischenlager der Staatlichen Museen zu Berlin, Germany; Rijksmuseum van Oudheden Rapenburg, Leiden, the Netherlands; The London British Museum & Petrie Museum, London, The United Kingdom; The Ancient Egyptian Mummy Tissue Bank, KNH Centre for Biomedical Egyptology, the University of Manchester, The United Kingdom; The Museo Egizio, Turin, Italy; The Archaeological Museum in Zagreb, Croatia; Museum für Völkerkunde, Burgdorf, Switzerland; Staatliches Museum Ägyptischer Kunst München (SMÄK), Munich, Germany; Musée d’Ethnographie, Neuchatel, Switzerland; Stiftsbibliothek, St. Gallen, Switzerland. Ethical approval was granted by these institutions for the retrieval of probable organic tissue associated with human remains. From this repository, 93 jars were selected for ancient DNA analysis testing (Table 1). Being fully encoded and older than 70 years (post-mortem), the sampled material does not require additional approval under Swiss law (Swiss Federal Act on Research involving human beings. Human Research Act, HRA art.1 and art.36; RS 810.30). Criteria for selection was based on whether there was any visible content in the jar and an estimation of the organic content of the jar through visual or electronic means such as planar X-ray, CT and MRI scanning. Visual inspection was the first step before any kind of imaging was done, and at this stage we excluded the empty jars. Conventional portable radiography, primarily on-site at museums or depots, was the preferred imaging method for practical reasons. Images of each jar were taken in two planes (anterior-posterior and lateral). The anterior side was defined as the side bearing the inscription (if present) for all imaging modalities. The aim of these imaging studies was mainly to better predict the expected quality of the samples. In the case of jars containing only small residual sediments or pulverized contents, the probability of obtaining samples containing actual organ components can be expected to be considerably lower than in the case of a canopic jar filled with solid contents. The selection criteria for planar X-rays were, on the one hand, the size of the canopic jars, given the available size of our X-ray detector of 43x35 cm, and, on the other hand, the coverage of a representative subsample of the entire range of possible canopic jar contents, concerning both the content level (from empty to full) and the degree of fragmentation of the contents (from powder to smaller to larger fragments or solid content). CT (5 canopic jars) and MRI (3 canopic jars) were primarily used to evaluate the feasibility and determine appropriate scan parameters, which we have published in detail elsewhere [(Eppenberger et al. 2018)](https://paperpile.com/c/K93WfD/1jdiQ). Sampling was performed with protective disposable Tyvek full-body coveralls and instruments decontaminated with UV light and bleach to avoid modern contamination. Depending on the integrity of the jar content, between 50-500 mg if material was sampled by scooping or punctuation with a biopsy needle and placed in sterile 2 mL Eppendorf tubes.

### **Processing of samples**

#### **Histology**

Sample preparation of mummified tissue for histology differs slightly from that of fresh tissue since mummified tissues are usually dehydrated. Direct fixation or direct embedding in paraffin will result in tissue sections with collapsed structures or without identifiable structures. Therefore, we rehydrated the tissue samples in phosphate-buffered saline (PBS) for 24 hours before fixation with 4% formaldehyde. Then, we embedded the samples in paraffin, followed by cutting two micron-thick sections and staining according to standard procedures. We viewed the stained histological sections with a conventional light microscope (40×, 100×, and 400× total magnification). The stainings used for the sample set included: Hematoxylin and eosin stain or haematoxylin and eosin stain (H&E stain or HE stain), Van Gieson's stain for differential staining of collagen and other connective tissue, Periodic acid–Schiff (PAS) to detect polysaccharides such as glycogen, and Gram staining to differentiate bacteria by the chemical and physical properties of their cell walls.

#### **DNA**

All DNA extractions and sequencing library preparations were performed in a clean room-type laboratory [(Knapp and Hofreiter 2010)](https://paperpile.com/c/K93WfD/ftu54) following standard anti-contamination protocols [(Cooper and Poinar 2000; Gilbert et al. 2005; Llamas et al. 2017)](https://paperpile.com/c/K93WfD/Vs4s4+pFzG7+or7Vg) with parallel non-template controls, in a laboratory dedicated solely to ancient DNA research at the Institute of Evolutionary Medicine, University of Zurich (Zurich, Switzerland). All PCR amplification, post-PCR, and Next Generation Sequencing (NGS) analysis were performed in a physically separated laboratory, and negative controls were added at different steps of the protocol (i.e., extraction and library prep steps). Three DNA extractions were performed, following the extraction method recommended for overcoming plant inhibitors based on CTAB (cetyltrimethylammonium bromide) and PTB (N-phenacylthiazolium bromide) [(Kistler 2012)](https://paperpile.com/c/K93WfD/Stb59), and a slight modification of the protocol by Dabney et al. (2013) [(Dabney et al. 2013)](https://paperpile.com/c/K93WfD/wstWf). Aliquots of 20 μl extract were converted into double-stranded Illumina libraries and double-indexed with sample-specific barcodes [(Meyer and Kircher 2010; Kircher, Sawyer, and Meyer 2012)](https://paperpile.com/c/K93WfD/aatUl+iBCTo) with parallel non-template controls. Sequencing libraries were generated following Meyer and Kircher 2010; Kircher, Sawyer, and Meyer (2012) with modifications. The re-amplification step was performed with 1 unit Herculase II DNA polymerase (Agilent), 5X Herculase II  reaction mix, 0.3 μM primers IS5 and IS6 [(Meyer and Kircher 2010)](https://paperpile.com/c/K93WfD/aatUl) and 4-7 μl library template with the following thermal profile: initial denaturation at 95°C for 2 min, 10 to 25 cycles of denaturation at 95°C for 30 sec, annealing at 60°C for 30 sec and elongation at 72°C for 30 sec, followed by a final elongation at 72°C for 5 min. Libraries were purified with MinElute spin columns (QIAGEN) following the manufacturer’s instructions. Quantitative PCR (qPCR) and analysis on an Agilent 2200 TapeStation were used to assess the quality and concentration of the libraries. Libraries were pooled equally and sequenced on one lane of a HiSeq2500 (Illumina) with paired-end 125 bp reads and v4 chemistry by the Functional Genomics Center Zurich (Zurich, Switzerland). Data is available upon submission under the SRA accession code SUB10667322 (NCBI).

#### **In-solution capture with DNA baits**

Based on histological data, a subset of samples was selected for further processing (Table 1) (Figs. 1-5). To optimize short-fragment recovery, we prepared single-stranded DNA libraries out of these samples [(Gansauge and Meyer 2019)](https://paperpile.com/c/K93WfD/KwRuE) and performed an enrichment for human mitochondrial DNA via bead capture hybridization [(Maricic, Whitten, and Pääbo 2010)](https://paperpile.com/c/K93WfD/vOlH7). Between 8 to 10 individually indexed libraries were pooled at a final concentration of 2 µg of DNA. For the blocking step, a blocking oligos mix composed of 500 µM of each blocking oligo primer Bio4, Bio6, Bio8 and Bio10 (Maricic, Whitten, and Pääbo 2010). Negative controls (extraction and library blanks) were pooled and enriched separately. After 48 h incubation at 65°C and several washing steps, the library molecules were eluted by NaOH melting and quantified by qPCR (LightCycler 480, Roche) following previously published methods (Maricic, Whitten, and Pääbo 2010). Prior to sequencing, sample pools and negative controls were combined equimolarly to a final concentration of 10nM. The resulting libraries were sequenced at a higher depth (5%) on an Illumina NextSeq500 machine using a Mid Output kit. Data is available upon submission under the SRA accession code SUB10667322 (NCBI).

## **Supplementary Note 2 - Analysis**

### **Metagenomic analysis - Methods**

Metagenomic screening of the samples was carried out with MALT which presents better identification power on aDNA sequencing data than other methods and databases that are based on partial genomes, e.g. 16S, ITS [(Vågene et al. 2018)](https://paperpile.com/c/K93WfD/WJxx1) using all complete bacterial, viral, and archaeal genomes in GenBank as a reference, [(Benson et al. 2013)](https://paperpile.com/c/K93WfD/oMSBk). We executed MALT with the following mapping parameters: reads with a minimum 85% identity (−−minPercentIdentity) were considered as a possible match to the reference; only nodes with minimum support of five reads (−−minSupport set to 5); BlastN mode and SemiGlobal alignment were applied and a top percent value of 1 (−−topPercent). The remaining parameters were set to default. MALT results were analyzed and visualized using MEGAN6 and phyloseq [(McMurdie and Holmes 2013; Huson et al. 2016)](https://paperpile.com/c/K93WfD/G6GwG+3clse).

### **Endogenous DNA - Methods**

To assess the levels of endogenous DNA in the samples, we used the EAGER pipeline  [(Peltzer et al. 2016)](https://paperpile.com/c/K93WfD/xp30a) to perform an alignment against both mitochondrial and whole human genomes. An important characteristic of ancient sequencing libraries is the occurrence of C to T substitution at the fragment ends [(Briggs et al. 2007)](https://paperpile.com/c/K93WfD/EeVM1). This is due to the post-mortem decay of DNA and can be used to authenticate ancient DNA. We selected the in-built function DamageProfiler in EAGER to estimate the frequency of damage at the 5’ and 3’ ends for ancient DNA authentication [(Neukamm, Peltzer, and Nieselt 2020)](https://paperpile.com/c/K93WfD/R5IX9).  Reads were adapter clipped, merged (suited for pair-end datasets), and quality trimmed using Clip&Merge [(Peltzer n.d.)](https://paperpile.com/c/K93WfD/Rr76m). For those read pairs that could not be merged because the overlap region was shorter than 10 nucleotides, or for which the corresponding read was removed during the combined adapter clipping and quality filtering step, the respective single-end reads were first trimmed at the 3’ end such that all bases have a Phred quality score of at least 20 and then mapped individually. The resulting reads for all samples were mapped to the human mitochondrial reference genome hg19 (NC_001807) and using the CircularMapper feature built in the EAGER pipeline, with an error rate (-n) of 0.2 to assure high specificity [(Peltzer n.d.)](https://paperpile.com/c/K93WfD/olnqE).

## **Supplementary Note 3 - Results**

### **Histological analysis - Results**

Figures 1 to 3 represent the only success with staining and identifying organic-like structures in the samples from the Canopic Jars. No organs or specific structures could be identified beyond a few indications of connective tissue or muscles, resin-like materials, and probable infiltration of fungal organisms.

### **Metagenomic analysis - Results**

When mapped to the National Center for Biotechnology Information (NCBI) taxonomy, the number of reads assigned dropped for all samples, limiting the estimation of the overall composition from major taxonomy groups. Of the main groups represented in the NCBI database (Bacteria, Fungi, Archaea, Virus), only the Bacteria branch presented enough resolution for reliable identification. Therefore, the metagenomic analysis presented here focuses only on bacterial groups. We estimated richness by calculating the bacterial alpha diversity using the observed, Chao1, and the Shannon indexes [(McMurdie and Holmes 2013)](https://paperpile.com/c/K93WfD/G6GwG), highlighting again both collection and sample variability; St.Gallen and Turin samples scored higher in all indexes. At the Phylum level, samples were mainly dominated by Proteobacteria (from 3% up to 100%), Actinobacteria (1% ~ 75%), and Firmicutes (0% ~ 92%). At the Family level, *Bacillaceae* was present to some extent (0.5% ~ 57%) in all samples, but especially in Manchester (0.5% ~ 39%), Munich (2% ~ 28%), and St. Gallen (8% ~ 9%) samples. *Mycobacteriaceae* was second in abundance, from less than 1% presence in some collections to up to 100% of all reads in the AM7194 sample from Berlin. *Pseudonocardiaceae* was present in every collection but more abundant in Manchester (3% ~ 22%), Munich (9% ~ 10%), Turin (2% ~ 12%), and Zagreb (5% ~ 15%). *Burkholderiaceae* was next, also present in all collections (0.1% ~ 19%) but dominating some of the samples from Berlin (79% ~ 100%) and s7007 samples from Turin (100%). *Clostridiaceae* was abundant in samples from Burgdorf (57% ~ 68%), in sample 3459A from Turin (53%), and present to some extent in other samples (0.1% ~ 12%). Other high abundances of note were *Staphylococcaceae* in Turin s7005 (22%), and *Lactobacillaceae* in Leiden HIIIss13 (25%).

At the genus level, the taxonomic characterization was more difficult and some samples (mostly from Leiden, Berlin, and Zagreb) had no satisfactory resolution. A total of 500,000 reads were mapped successfully to *Oceanobacillus* in Leiden AT1C, around 380,000 reads in Manchester 509.2, and 80,000 reads in Manchester 509.1. These reads mapped to the species *Oceanobacillus iheyensis*, a surprising result considering this species is a deep-sea extremely halotolerant and alkaliphilic bacteria isolated from a depth of 1050 m on the Iheya Ridge [(Lu, Nogi, and Takami 2001)](https://paperpile.com/c/K93WfD/w5hot). Second in abundance was *Mycobacterium* with 480,000 reads mapping in Leiden AT1C, 220,000 reads in Manchester 509.2, and 150,000 reads in Turin c3214.4. When analyzed to the species level, no clear resolution was made between different members of the *Mycobacterium* genus, so we tested mapping against the taxa with most reads assigned: *M. kansasii* and *M. tuberculosis.* Sample AT1C from Leiden had 37,224 reads mapping to *M. kansasii* and 30,271 reads to *M. tuberculosis*, but both sets of reads had very low damage frequency profiles of 0.06%. Manchester 509.2 had 1,784 reads for *M. kansasii* and 1,409 for *M. tuberculosis*, with no observable damage. Members of Mycobacteria are widespread organisms and many are saprophytic, so this identification is likely to be due to environmental contamination and tissue decay rather than the presence of pathogens. The next genus in abundance was *Streptomyces*, again Leiden AT1C with the most number of reads assigned (360,000 reads) followed by Turin c3214.4 (30,000), St. Gallen 2 (25,000), and Manchester 509.01 (18,000). The *Streptomyces* genus has an extensive number of members and all the reads mapped equally distributed to different species without a clear taxonomical distinction. *Streptomyces* species are typically found in soil and decaying matter and are very resistant to adverse conditions due to their ability to form spores, which lends further evidence supporting an environmental intrusion. Genus *Clostridium* was particularly abundant in the Burgdorf samples (305,000 reads in sample 3 and 90,000 reads in sample 2), but with just traces in the rest of the collections (few hundreds up to 25,000); from these samples, *C. botulinum* was the most abundant species. The causative agent of botulism is also an environmental spore-forming bacteria ubiquitously distributed in soils and aquatic sediments and thrives in decomposing organic matter [(Figueiredo et al. 2020)](https://paperpile.com/c/K93WfD/skEZj). Next was *Burkholderia*, another genus abundant in soil, with more than 300,000 reads in Leiden AT1c.1, Turin 3495C with 76,000, and St. Gallen 2 with 28,000. Then was the genus *Virgibacillus*, known to be spore-forming and mostly isolated from saline environments, having 160,000 reads in Leiden AT1c.1, 130,000 reads in Turin s7005, and 509.02 20,000 reads in Manchester. The taxonomy was not resolved at the species level; however, *B. subtilis* had an abundance of 51,000 reads. *B. subtilis,* also known as the hay bacillus or grass bacillus, is found in soil and the gastrointestinal tract of ruminants and humans. Then was *Corynebacterium* (176,000 reads in St. Gallen 2, 30,000 reads in Leiden AT1c.1), which was completely dominated by *C. halotolerans*, an environmental halophile. Next in abundance was *Lactobacillus*, very present in both Leiden AT1c.1 and Leiden HIIIss12 (119,000 reads and 111,000 reads respectively), with *L. johnsonii* and *L. reuteri*, both typically found in human and animal gastrointestinal tracts, but also in food, vegetation, and sewage environments. Genera that were found in the blanks and that are known to be common residual contaminants in the reagents were not taken into account, mainly *Pseudomonas* and certain species of *Burkholderia* (Salter et al. 2014).

### **Endogenous human DNA - Results**

Screening of the shallow sequencing data revealed no presence of reads that could be mapped against the human mitochondrial DNA or human nuclear DNA, raising the question of whether there was human tissue in the jars at all. Based on the positive histological analysis of the Berlin and Zagreb samples, we applied an enrichment approach to optimize the recovery of human mtDNA. Following hybridization, only three out of 14 samples had more than 1000 reads: Zagreb 607 (with 8,081 reads) and Berlin AM7170/3 (4398 reads), and Berlin AM7179 (3,142 reads). The contamination estimation and haplogroup assignment characterized sample Zagreb 607 as European haplogroup H (92.60% quality, contamination estimated to be less than 1%), and sample Berlin AM7179 was assigned to haplogroup R0a1 (70% quality, contamination estimated to be less than 1%), characteristic of Middle-East populations [(Renaud et al. 2015; Weissensteiner et al. 2016)](https://paperpile.com/c/K93WfD/nPKAu+Du5Ju). The amount and quality of the reads for the rest of the samples was not enough for a confident estimation of the haplogroup.

#

#

#

## **Supplementary tables**

**Table 1:** List of samples screened in this study, organised by collection and indicating the supposed tissue the jar may be containing based on the Egyptological context. In bold are the samples that were selected for single-strand library preparation.

| **Collection** | **ID** | **Type** | **Weight** | **Supposed tissue content** |
| --- | --- | --- | --- | --- |
| Munich | ÄS_0026_1 | Powder core | 200 mg | Liver (Hapy) |
| Munich | ÄS_0026_2 | Powder core | 200 mg | Liver (Hapy) |
| Munich | ÄS_0026_3 | Powder core | 200 mg | Liver (Hapy) |
| Munich | ÄS_0026_4 | Powder core | 200 mg | Liver (Hapy) |
| Munich | ÄS_0026_5 | Powder core | 200 mg | Liver (Hapy) |
| Munich | ÄS_0026_6 | Powder core | 200 mg | Liver (Hapy) |
| Munich | ÄS_0026_7 | Powder core | 200 mg | Liver (Hapy) |
| Munich | ÄS_0026_8 | Powder core | 200 mg | Liver (Hapy) |
| Munich | ÄS_0026_9 | Powder core | 100 mg | Liver (Hapy) |
| Burgdorf | BU_53_07668_1 | Surface | 50 mg | Intestines (Qebekhsenuef) |
| Burgdorf | BU_53_07668_2 | Surface | 50 mg | Intestines (Qebekhsenuef) |
| Burgdorf | BU_53_07668_3 | Surface | 50 mg | Intestines (Qebekhsenuef) |
| Burgdorf | BU_53_07668_4 | Surface | 50 mg | Intestines (Qebekhsenuef) |
| St. Gallen | 1 | Sawdust | 200 mg | Unknown |
| St. Gallen | 2 | Sawdust | 200 mg | Unknown |
| Turin | A | Powder | 100 mg | Unknown |
| Turin | C | Powder | 100 mg | Unknown |
| Turin | c3211/3 | Surface | 34 mg | Unknown |
| Turin | c3212 | Scoop | 185 mg | Intestines (Qebekhsenuef) |
| Turin | c3214/1_1 | Powder | 32 mg | Lung (Imset) |
| Turin | c3214/1_2 | Lump | 79 mg | Lung (Imset) |
| Turin | c3214/3_1 | Surface | 125 mg | Stomach (Duamutef) |
| Turin | c3214/3_2 | Scoop | 280 mg | Stomach (Duamutef) |
| Turin | c3215/4_1 | Scoop | 50 mg | Stomach (Duamutef) |
| Turin | c3215/4_2 | Scoop | 35 mg | Stomach (Duamutef) |
| Turin | c3217/11 | Powder | 150 mg | Liver (Hapy) |
| Turin | c3308 | Scoop | 235 mg | Lung (Imset) |
| Turin | c3304_1 | Biopsy | 10 mg | Stomach (Duamutef) |
| Turin | c3304_2 | Bandage | 15 mg | Stomach (Duamutef) |
| Turin | c3457/1_1 | Surface | 15 mg | Liver (Hapy) |
| Turin | c3457/1_2 | Bandage | 10 mg | Liver (Hapy) |
| Turin | c3457/2_1 | Bandage | 15 mg | Unknown |
| Turin | c3457/2_2 | Surface | 15 mg | Unknown |
| Turin | c3461 | Powder | 44 mg | Unknown |
| Turin | c3464_1 | Bandage | 15 mg | Intestines (Qebekhsenuef) |
| Turin | c3464_2 | Powder | 65 mg | Intestines (Qebekhsenuef) |
| Turin | c3465a_1 | Bandage | 30 mg | Unknown |
| Turin | c3465a_2 | Powder | 90 mg | Unknown |
| Turin | c3465b_1 | Bandage | 10 mg | Unknown |
| Turin | c3465b_2 | Surface | 20 mg | Unknown |
| Turin | c3466 | Bandage | 10 mg | Unknown |
| Turin | c3567 | Bandage | 15 mg | Unknown |
| Turin | c3471/2 | Powder | 50 mg | Unknown |
| Turin | s4306_1 | Powder | 30 mg | Unknown |
| Turin | s4306_2 | Lump | 20 mg | Unknown |
| Turin | s4308 | Surface | 40 mg | Unknown |
| Turin | s7005_1 | Bandage | 30 mg | Unknown |
| Turin | s7005_2 | Sand | 325 mg | Unknown |
| Turin | s7007 | Powder | 57 mg | Unknown |
| Turin | s7008 | Powder | 65 mg | Unknown |
| Leiden | AAL1c | Lump | 5000 mg | Liver (Hapy) |
| Leiden | AAL1d_1 | Lump | 2000 mg | Lung (Imset) |
| Leiden | AAL1d_2 | Powder | 1000 mg | Lung (Imset) |
| Leiden | AAL1e | Lump | 2500 mg | Intestines (Qebekhsenuef) |
| Leiden | AAL3a | Scoop | 350 mg | Unknown |
| Leiden | AAL3b | Core powder | 150 mg | Unknown |
| Leiden | AAL3e | Scoop | 350 mg | Unknown |
| Leiden | AAL8 | Powder | 350 mg | Unknown |
| Leiden | AAL9a | Lump | 4000 mg | Unknown |
| Leiden | AAL9b | Lump | 5000 mg | Unknown |
| Leiden | AT1c_1 | Lump | 4000 mg | Unknown |
| Leiden | AT1c_2 | Bandage | 15 mg | Unknown |
| Leiden | AT1c_3 | Powder | 230 mg | Unknown |
| Leiden | CI275_1 | Lumps | 120 mg | Intestines (Qebekhsenuef) |
| Leiden | CI275_2 | Core powder | 3000 mg | Intestines (Qebekhsenuef) |
| Leiden | HIIIss12 | Powder | 81 mg | Unknown |
| Leiden | HIIIss13 | Core powder | 330 mg | Unknown |
| Manchester | F6.52.01 | Powder | 70 mg | Unknown |
| Manchester | F6 509.01 | Powder | 70 mg | Unknown |
| Manchester | F6 509.02 | Powder | 80 mg | Unknown |
| Manchester | F6 566.01 | Powder | 70 mg | Unknown |
| Manchester | 474-01 | Powder | 65 mg | Unknown |
| Manchester | 474-10 | Powder | 80 mg | Unknown |
| Manchester | 474-11 | Powder | 75 mg | Unknown |
| Manchester | 474-18 | Powder | 60 mg | Unknown |
| **Zagreb** | **610** | **Powder** | **90 mg** | **Unknown** |
| **Zagreb** | **617** | **Powder** | **100 mg** | **Unknown** |
| **Zagreb** | **622-1** | **Powder** | **85 mg** | **Unknown** |
| **Zagreb** | **621** | **Powder** | **100 mg** | **Unknown** |
| **Zagreb** | **677** | **Powder** | **95 mg** | **Unknown** |
| **Berlin** | **ÄM 7170/03** | **Powder** | **100 mg** | **Unknown** |
| **Berlin** | **ÄM 7184/03** | **Powder** | **100 mg** | **Intestines (Qebekhsenuef)** |
| **Berlin** | **ÄM 7185/03** | **Powder** | **100 mg** | **Lung (Imset)** |
| **Berlin** | **ÄM 7186** | **Powder** | **100 mg** | **Liver (Hapy)** |
| **Berlin** | **ÄM 7187** | **Powder** | **100 mg** | **Stomach (Duamutef)** |
| **Berlin** | **ÄM 16459** | **Powder** | **100 mg** | **Unknown** |
| **Berlin** | **ÄM 8428** | **Powder** | **100 mg** | **Unknown** |
| **Berlin** | **ÄM 7199** | **Powder** | **100 mg** | **Intestines (Qebekhsenuef)** |
| **Berlin** | **ÄM 7171** | **Small lump** | **<100 mg** | **Stomach (Duamutef)** |
| **Berlin** | **ÄM 7165** | **Powder/sand** | **100 mg** | **Unknown** |
| **Berlin** | **ÄM 7183** | **Powder/sand** | **100 mg** | **Stomach (Duamutef)** |
| **Berlin** | **ÄM 7653** | **Powder** | **100 mg** | **Intestines (Qebekhsenuef)** |
| **Berlin** | **ÄM 7194** | **Bundle** | **100 mg** | **Unknown** |
| **Berlin** | **ÄM 7170/04** | **Lump** | **100 mg** | **Unknown** |

**Table 2:** Mitochondrial reads present in the samples after the hybridization enrichment protocol. Z = Zagreb, B = Berlin, S = Surface, P = Powder, EnDNA = Endogenous DNA content, CF = cluster factor, DMG = Damage, NA = no data.

| **ID** | **Museum ID** | **Reads** | **EnDNA (%)** | **CF** | **> 1X (%)** | **> 5X (%)** | **DMG (%)** |
| --- | --- | --- | --- | --- | --- | --- | --- |
| ZH0580 | 1(610-2)_Z | 132 | 0.282 | 49.31 | 21.69 | 4.27 | NA |
| ZH0582 | 3(622-1)_Z | 29 | 0.087 | 6.06 | 6.53 | 0.88 | NA |
| ZH0584 | 5(607)S_ Z | 112 | 0.718 | 46.61 | 20.91 | 2.27 | NA |
| ZH0585 | 5(607)P_Z | 8081 | 2.083 | 35.88 | 100 | 99.73 | 4 |
| ZH0586 | AM7165_B | 299 | 2.398 | 15.43 | 47.81 | 4.91 | NA |
| ZH0587 | AM7170/3_B | 4398 | 2.62 | 25.35 | 99.08 | 87.58 | 10 |
| ZH0588 | AM7170/4_B | 363 | 7.304 | 57.94 | 34.62 | 11.03 | NA |
| ZH0589 | AM7179_ B | 3142 | 0.976 | 51.15 | 96.21 | 75.33 | 9 |
| ZH0590 | AM7184/3_B | 123 | 1.268 | 81.26 | 13.6 | 3.71 | NA |
| ZH0591 | AM7185_B | 575 | 0.947 | 21.13 | 58.74 | 15.74 | NA |
| ZH0592 | AM7194_B | 904 | 0.126 | 50.53 | 70.43 | 28.96 | NA |
| ZH0593 | AM7653.1_B | 523 | 3.655 | 118.03 | 38.35 | 15.84 | NA |
| ZH0594 | AM7653.2_B | 784 | 3.031 | 48.03 | 59.79 | 27.62 | NA |
| ZH0595 | AM7653.3_B | 582 | 5.273 | 17.47 | 62.55 | 17.97 | NA |

**Table 3:** List of probable taxa belonging to the human gut microbiome identified in the canopic jar samples.

| **Species** | **No Reads** | **>1X (%)** | **Damage (%)** | **Sample** |
| --- | --- | --- | --- | --- |
| *Alistipes finegoldii* | 6012 | 1.24 | 11.0 | AT1c R, Leiden |
| *Bifidobacterium longum* | 11287 | 2.84 | 11.0 | AT1c R, Leiden |
| *Clostridium botulinum* | 28002 | 3.19 | 5.5 | Burgdorf No.3 |
| *Desulfovibrio fairfieldensis* | 16866 | 1.67 | 8.0 | AT1c R, Leiden |
| *Faecalibacterium prausnitzii* | 8633 | 1.51 | 10.0 | AT1c R, Leiden |
| *Klebsiella pneumoniae* | 11718 | 1.85 | 7.0 | AT1c R, Leiden |
| *Mycobacterium kansasii* | 37224 | 6.52 | 8.0 | AT1c R, Leiden |
| *Mycobacterium tuberculosis* | 30271 | 7.42 | 8.0 | AT1c R, Leiden |
| *Oceanobacillus iheyensis* | 21820 | 1.7 | 8.0 | AT1c R, Leiden |

##

## **Supplementary figures**

**Figure 1:** Sample Zagreb 617, Surface sampling. HE-staining showing resin-like material.


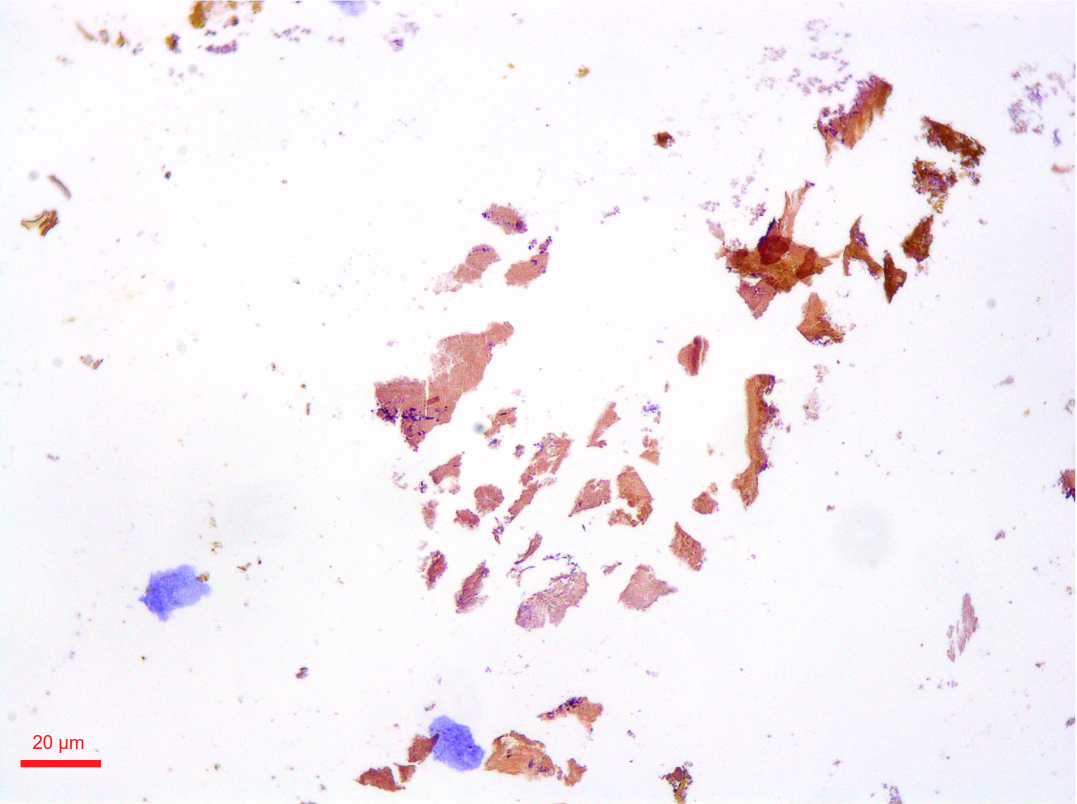


**Figure 2:** Sample 622-1, perforated sampling with biopsy needle. PAS-Staining showing infiltrating fungi (arrow).


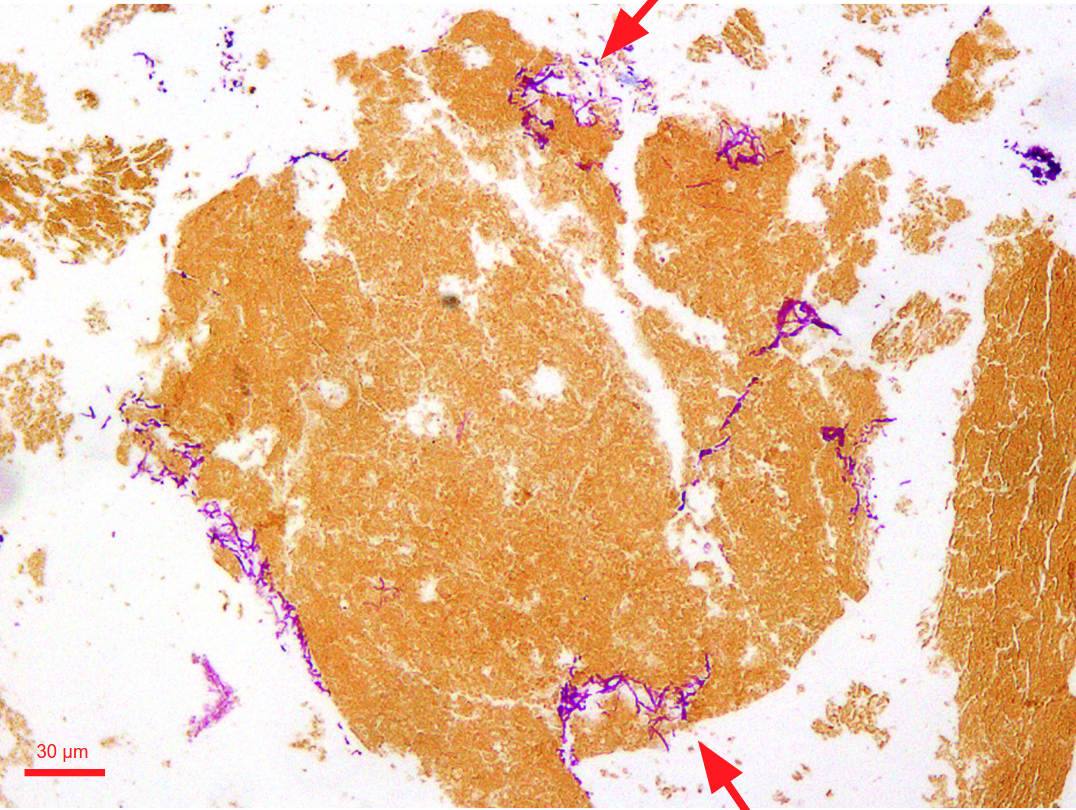


**Figure 4:** Sample 622-1, perforated sampling with biopsy needle. HE-Staining showing resin-like material (arrow).


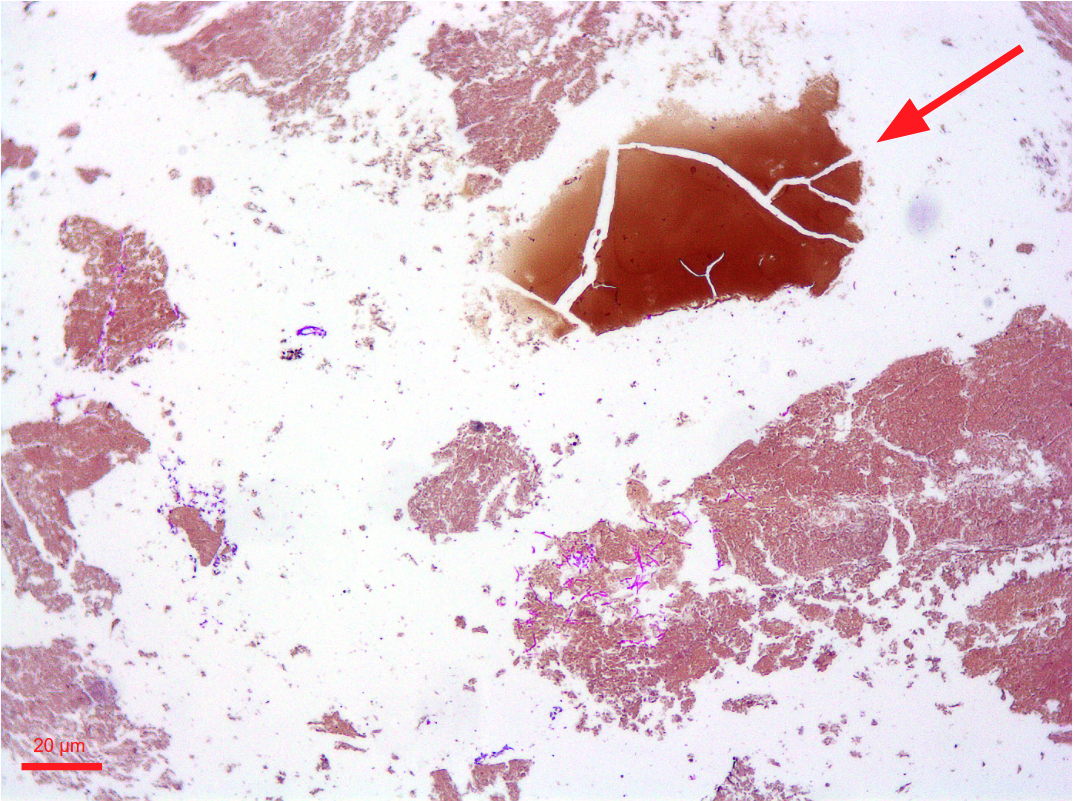


**Figure 5:** Damage pattern for the 8081 reads in sample Zagreb 607.


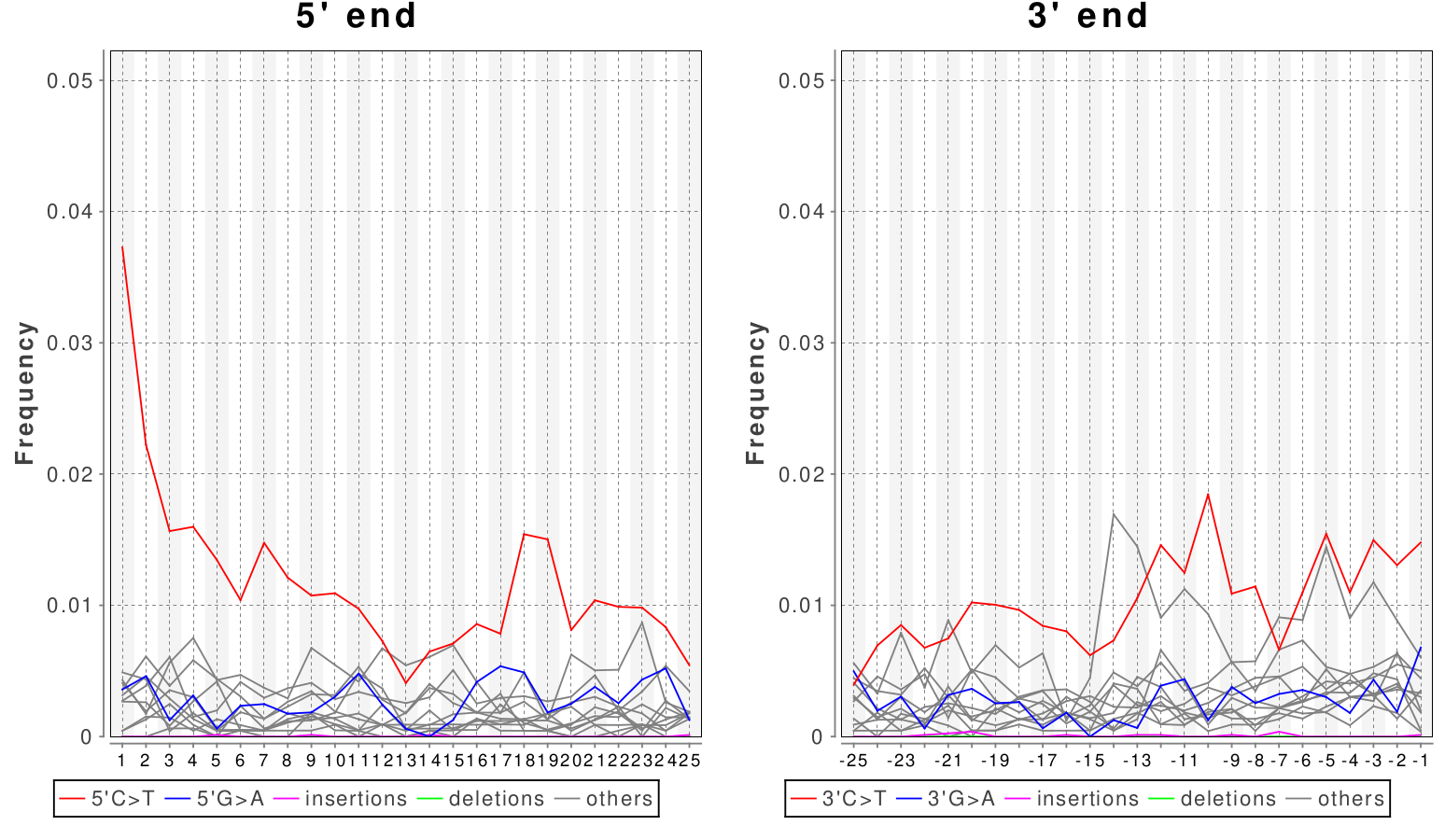


**Figure 6:** Damage pattern for the 4398 reads in sample Berlin AM7170/3.
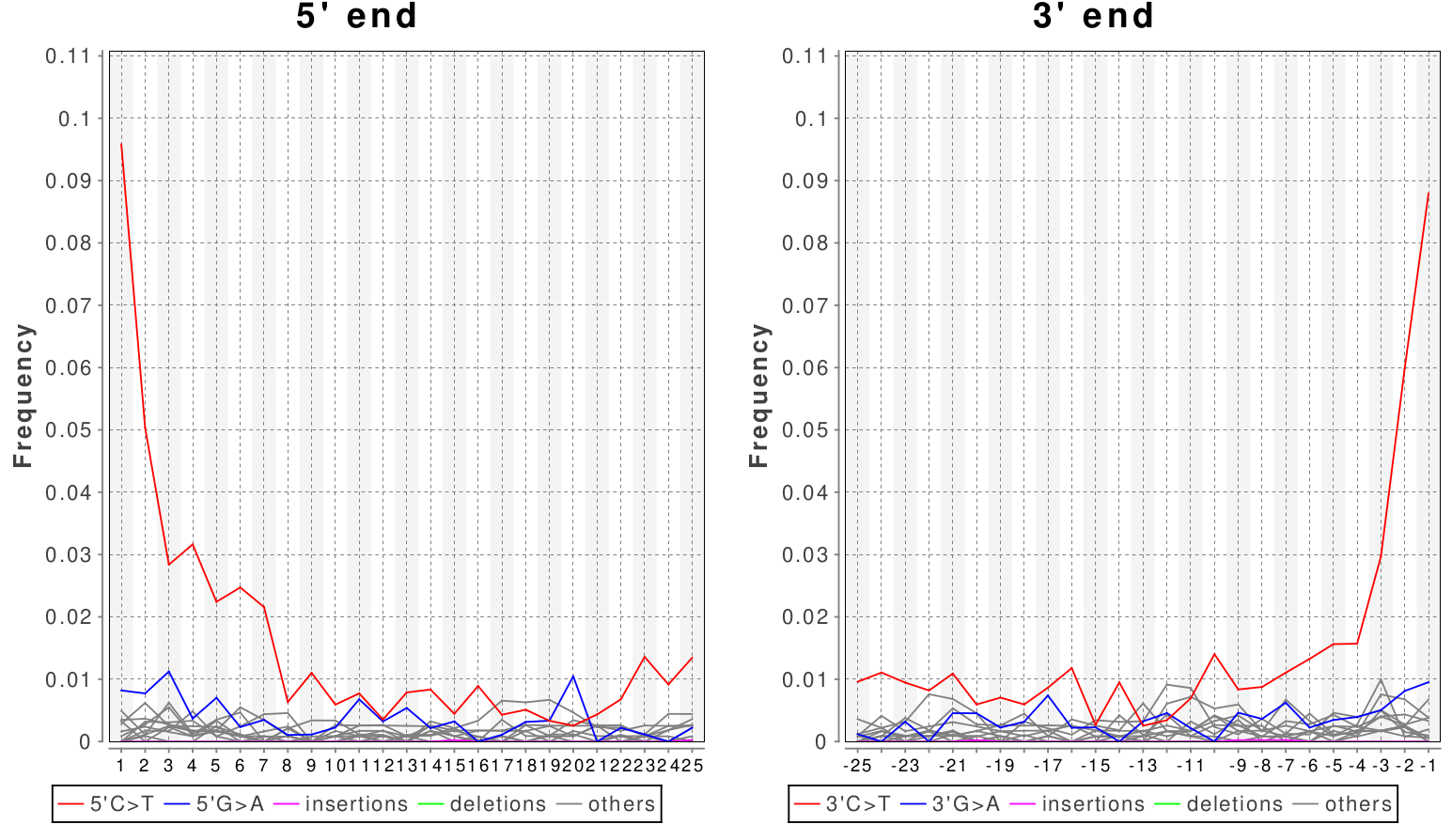


**Figure 7:** Damage pattern for the 3142 reads in sample Berlin AM7179.


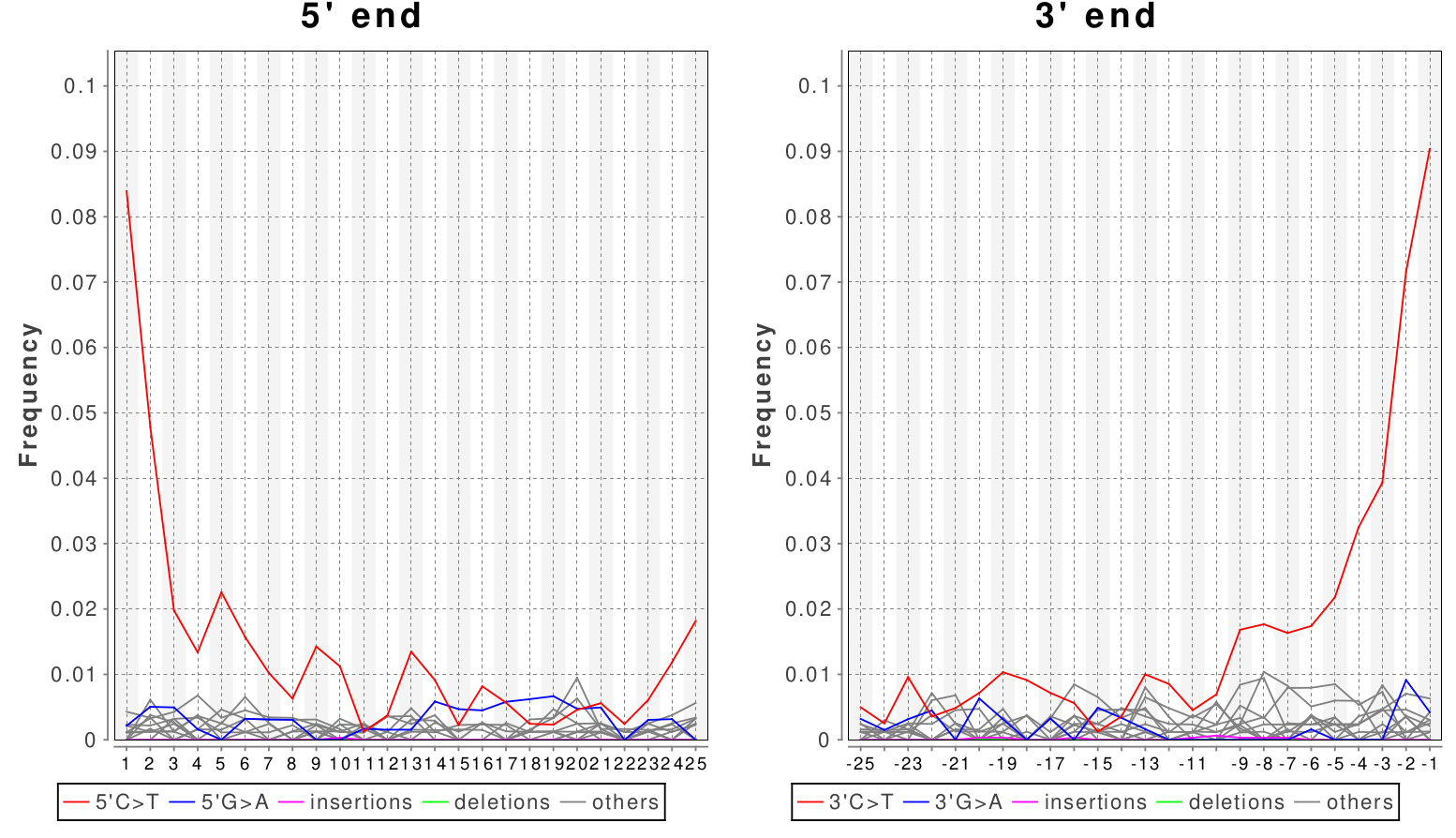


**Figure 8:** Most abundant families present in the samples, expressed in percentage and grouped by collection - and blanks. **
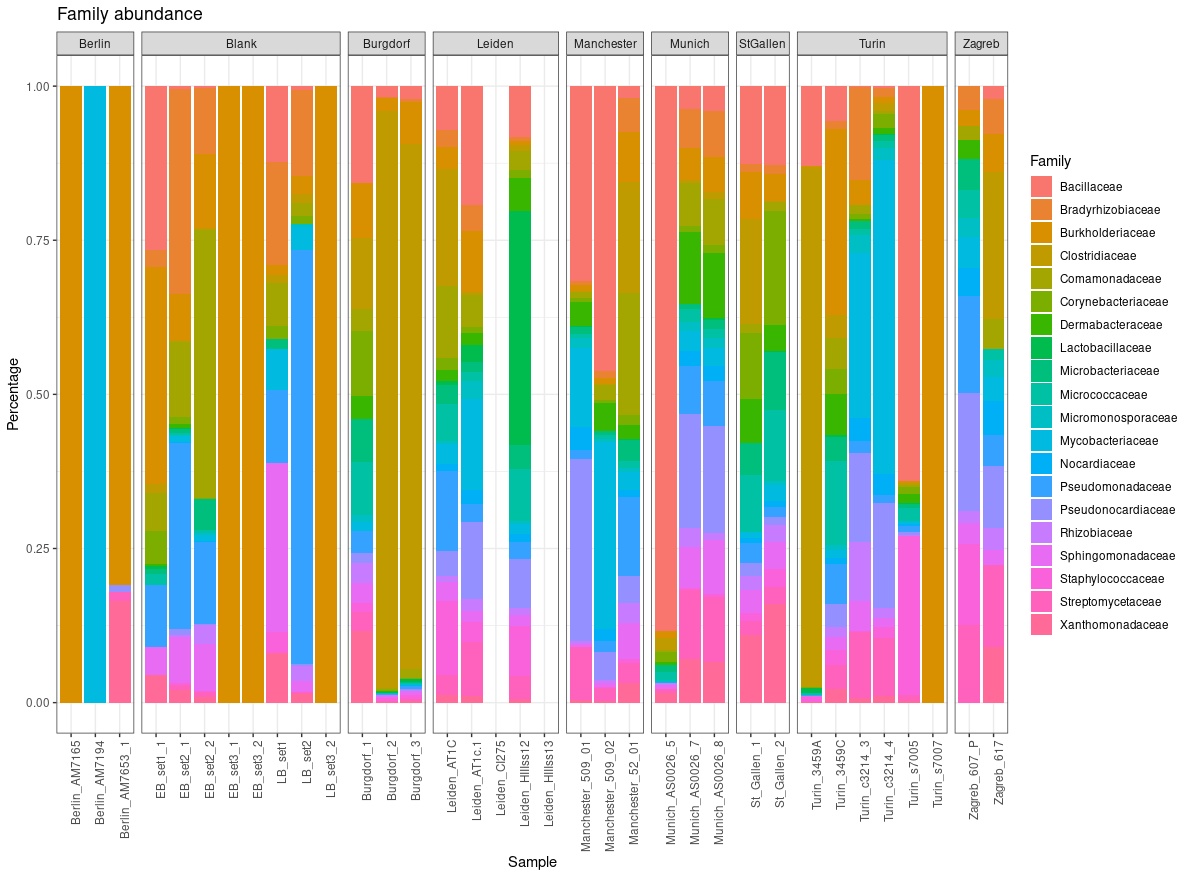
**

## **References**

[Benson, Dennis A., Mark Cavanaugh, Karen Clark, Ilene Karsch-Mizrachi, David J. Lipman, James Ostell, and Eric W. Sayers. 2013. “GenBank.” *Nucleic Acids Research* 41 (Database issue): D36–42.](http://paperpile.com/b/K93WfD/oMSBk)

[Briggs, Adrian W., Udo Stenzel, Philip L. F. Johnson, Richard E. Green, Janet Kelso, Kay Prüfer, Matthias Meyer, et al. 2007. “Patterns of Damage in Genomic DNA Sequences from a Neandertal.” *Proceedings of the National Academy of Sciences of the United States of America* 104 (37): 14616–21.](http://paperpile.com/b/K93WfD/EeVM1)

[Cooper, Alan, and Hendrik N. Poinar. 2000. “Ancient DNA: Do It Right or Not at All.” *Science* 289 (5482): 1139–1139.](http://paperpile.com/b/K93WfD/Vs4s4)

[Dabney, Jesse, Michael Knapp, Isabelle Glocke, Marie-Theres Gansauge, Antje Weihmann, Birgit Nickel, Cristina Valdiosera, et al. 2013. “Complete Mitochondrial Genome Sequence of a Middle Pleistocene Cave Bear Reconstructed from Ultrashort DNA Fragments.” *Proceedings of the National Academy of Sciences of the United States of America* 110 (39): 15758–63.](http://paperpile.com/b/K93WfD/wstWf)

[Eppenberger, Patrick E., Mislav Cavka, Michael E. Habicht, Francesco M. Galassi, and Frank Rühli. 2018. “Radiological Findings in Ancient Egyptian Canopic Jars: Comparing Three Standard Clinical Imaging Modalities (x-Rays, CT and MRI).” *European Radiology Experimental* 2 (December): 12.](http://paperpile.com/b/K93WfD/1jdiQ)

[Figueiredo, Guilherme Grodzki Oliveira, Valéria Rosa Lopes, Tales Romano, and Marcela Candido Camara. 2020. “Chapter 22 - Clostridium.” In *Beneficial Microbes in Agro-Ecology*, edited by N. Amaresan, M. Senthil Kumar, K. Annapurna, Krishna Kumar, and A. Sankaranarayanan, 477–91. Academic Press.](http://paperpile.com/b/K93WfD/skEZj)

[Gansauge, Marie-Theres, and Matthias Meyer. 2019. “A Method for Single-Stranded Ancient DNA Library Preparation.” *Methods in Molecular Biology*  1963: 75–83.](http://paperpile.com/b/K93WfD/KwRuE)

[Gilbert, M. Thomas P., Hans-Jürgen Bandelt, Michael Hofreiter, and Ian Barnes. 2005. “Assessing Ancient DNA Studies.” *Trends in Ecology & Evolution* 20 (10): 541–44.](http://paperpile.com/b/K93WfD/pFzG7)

[Huson, Daniel H., Sina Beier, Isabell Flade, Anna Górska, Mohamed El-Hadidi, Suparna Mitra, Hans-Joachim Ruscheweyh, and Rewati Tappu. 2016. “MEGAN Community Edition - Interactive Exploration and Analysis of Large-Scale Microbiome Sequencing Data.” *PLoS Computational Biology* 12 (6): e1004957.](http://paperpile.com/b/K93WfD/3clse)

[Kircher, Martin, Susanna Sawyer, and Matthias Meyer. 2012. “Double Indexing Overcomes Inaccuracies in Multiplex Sequencing on the Illumina Platform.” *Nucleic Acids Research* 40 (1): e3.](http://paperpile.com/b/K93WfD/iBCTo)

[Kistler, Logan. 2012. “Ancient DNA Extraction from Plants.” In *Ancient DNA: Methods and Protocols*, edited by Beth Shapiro and Michael Hofreiter, 71–79. Totowa, NJ: Humana Press.](http://paperpile.com/b/K93WfD/Stb59)

[Knapp, Michael, and Michael Hofreiter. 2010. “Next Generation Sequencing of Ancient DNA: Requirements, Strategies and Perspectives.” *Genes* 1 (2): 227–43.](http://paperpile.com/b/K93WfD/ftu54)

[Llamas, Bastien, Guido Valverde, Lars Fehren-Schmitz, Laura S. Weyrich, Alan Cooper, and Wolfgang Haak. 2017. “From the Field to the Laboratory: Controlling DNA Contamination in Human Ancient DNA Research in the High-Throughput Sequencing Era.” *STAR: Science & Technology of Archaeological Research* 3 (1): 1–14.](http://paperpile.com/b/K93WfD/or7Vg)

[Lu, J., Y. Nogi, and H. Takami. 2001. “Oceanobacillus Iheyensis Gen. Nov., Sp. Nov., a Deep-Sea Extremely Halotolerant and Alkaliphilic Species Isolated from a Depth of 1050 M on the Iheya Ridge.” *FEMS Microbiology Letters* 205 (2): 291–97.](http://paperpile.com/b/K93WfD/w5hot)

[Maricic, Tomislav, Mark Whitten, and Svante Pääbo. 2010. “Multiplexed DNA Sequence Capture of Mitochondrial Genomes Using PCR Products.” *PloS One* 5 (11): e14004.](http://paperpile.com/b/K93WfD/vOlH7)

[McMurdie, Paul J., and Susan Holmes. 2013. “Phyloseq: An R Package for Reproducible Interactive Analysis and Graphics of Microbiome Census Data.” *PloS One* 8 (4): e61217.](http://paperpile.com/b/K93WfD/G6GwG)

[Meyer, Matthias, and Martin Kircher. 2010. “Illumina Sequencing Library Preparation for Highly Multiplexed Target Capture and Sequencing.” *Cold Spring Harbor Protocols* 2010 (6): db.prot5448.](http://paperpile.com/b/K93WfD/aatUl)

[Neukamm, Judith, Alexander Peltzer, and Kay Nieselt. 2020. “DamageProfiler: Fast Damage Pattern Calculation for Ancient DNA.” *Cold Spring Harbor Laboratory*. https://doi.org/](http://paperpile.com/b/K93WfD/R5IX9)[10.1101/2020.10.01.322206](http://dx.doi.org/10.1101/2020.10.01.322206)[.](http://paperpile.com/b/K93WfD/R5IX9)

[Peltzer, Alexander. n.d. *CircularMapper*. Github. Accessed January 26, 2021a.](http://paperpile.com/b/K93WfD/olnqE) <https://github.com/apeltzer/CircularMapper>[.](http://paperpile.com/b/K93WfD/olnqE)

[———. n.d. *ClipAndMerge*. Github. Accessed January 26, 2021b.](http://paperpile.com/b/K93WfD/Rr76m) <https://github.com/apeltzer/ClipAndMerge>[.](http://paperpile.com/b/K93WfD/Rr76m)

[Peltzer, Alexander, Günter Jäger, Alexander Herbig, Alexander Seitz, Christian Kniep, Johannes Krause, and Kay Nieselt. 2016. “EAGER: Efficient Ancient Genome Reconstruction.” *Genome Biology* 17 (March): 60.](http://paperpile.com/b/K93WfD/xp30a)

[Renaud, Gabriel, Viviane Slon, Ana T. Duggan, and Janet Kelso. 2015. “Schmutzi: Estimation of Contamination and Endogenous Mitochondrial Consensus Calling for Ancient DNA.” *Genome Biology* 16 (October): 224.](http://paperpile.com/b/K93WfD/nPKAu)

[Vågene, Åshild J., Alexander Herbig, Michael G. Campana, Nelly M. Robles García, Christina Warinner, Susanna Sabin, Maria A. Spyrou, et al. 2018. “Salmonella Enterica Genomes from Victims of a Major Sixteenth-Century Epidemic in Mexico.” *Nature Ecology & Evolution* 2 (3): 520–28.](http://paperpile.com/b/K93WfD/WJxx1)

[Weissensteiner, Hansi, Dominic Pacher, Anita Kloss-Brandstätter, Lukas Forer, Günther Specht, Hans-Jürgen Bandelt, Florian Kronenberg, Antonio Salas, and Sebastian Schönherr. 2016. “HaploGrep 2: Mitochondrial Haplogroup Classification in the Era of High-Throughput Sequencing.” *Nucleic Acids Research* 44 (W1): W58–63.](http://paperpile.com/b/K93WfD/Du5Ju)
